# Editorial: Exploring social stratification dynamics: insights from longitudinal survey data

**DOI:** 10.3389/fsoc.2025.1706477

**Published:** 2025-10-29

**Authors:** Robin Tillmann, Florence Lebert, Ursina Kuhn, Monica Budowski

**Affiliations:** ^1^Swiss Centre of Expertise in the Social Sciences FORS, Lausanne, Switzerland; ^2^University of Fribourg, Fribourg, Switzerland

**Keywords:** panel data, social mobility, social stratification, life course, education, labor market, political attitudes

## Introduction

This Research Topic compiles articles based on longitudinal survey data, which are particularly useful for examining the dynamics of various facets of social stratification. Long-running panel studies, such as the Swiss Household Panel, are particularly well-suited to understanding processes of mobility and inertia. They make it possible to (a) measure and analyze social change; (b) distinguish between permanent and transitory characteristics of a given phenomenon; and (c) study both inter- and intragenerational patterns of phenomena such as poverty, income dynamics, health conditions and practices, or political positioning. Additionally, they enable researchers to establish (robust) causal relationships between social phenomena rather than hypothesizing causal explanations based on associations. Moreover, in the case of household panels, they allow for intra-household studies, such as the mutual influence of household members' attitudes and behaviors over time and their impact on social stratification. They also make it possible to disentangle individual and household contextual causes for mobility and inertia and reveal the extent to which factors of change or inertia may change over time, due to changes in legislation or social policies.

This Research Topic aims to make use of the advantages of longitudinal data for stratification research. Questions of interest for the issue include: What are the stratifying factors over time? What dynamics exist? Have novel factors been identified (e.g., segregation, networks, social policies, and political behavior) that impact stratification? What role do intra-household factors play? The contributions of this Research Topic are based on various longitudinal studies: the Swiss Household Panel (SHP), the Italian Lives Survey (ITA.LI), the Cologne High School Panel (CHiSP), and the Transitions from Education to Employment data (TREE) collected in Switzerland.

## Contributions to this Research Topic

This Research Topic contains eight articles. The article by Birkelbach et al. is devoted to an educationally privileged group of high school students. The strength of the study is that it follows a cohort over their whole occupational career—from age 16–66. It analyses the effect of given vs. controllable starting conditions at age 16 on occupational success over the life course and shows the mechanisms of educational selection. The authors conclude that the starting conditions lose their impact over time and that occupational careers stabilize autonomously.

Nennstiel and Becker examine occupational prestige trajectories in Switzerland from 1946 to 2023, analyzing how macro trends such as modernization, unemployment, and cohort size shape individual careers. They show that younger generations benefit from structural changes, such as the expansion of education and the importance of the service economy. Over time, education has gained importance, mitigating negative structural effects. While men's prestige trajectories are more affected by economic fluctuations, women's depend more strongly on educational investments.

Poltier explores how early labor market experiences shape economic attitudes. Specifically, he examines how exposure to foreign demand affects the social spending preferences of young people in their early years in the labor market. He demonstrates that individuals entering the labor market in sectors that rely on exports tend to adopt a more critical attitude toward social spending. He concludes that although labor market entrants self-select into occupations based on their political preferences, their early labor market experiences lead them to adjust these preferences.

Bussi et al. examine the influence of social class of origin on labor market entry in Italy across different birth cohorts, genders, and regions, considering the mediating influence of education. They show that, due to prolonged education, individuals with higher social backgrounds enter the labor market later in life than individuals from lower-status families. Although education somewhat reduces class-based disparities, social origin continues to play a major role in shaping labor market entry in Italy.

Edler and Hadjar explore educational trajectories within and beyond the core schooling phase, considering later achievements and higher vocational qualifications. They uncover disparities in educational patterns linked to social origin, migration background, and gender. Their findings indicate a lengthening of the education phase over time, accompanied by a rise in higher education and vocational training. While inequalities persist, vocationally oriented pathways can help offset early disadvantages and reduce educational inequality.

Gomensoro et al. study the intergenerational occupational status transfer in two Swiss school-leaving cohorts (2000 and 2016) completing vocational education and training (VET) programs at the upper-secondary level of education. Using the Occupational Earnings Potential scale to analyze the association between the occupational status of parents and children, they find that, overall, status transmission remains stable, despite a reduced impact of lower-secondary tracking. Institutional changes have not diminished social inequality, leading the authors to advocate for greater educational equity beyond the formal permeability of Switzerland's highly stratified school system.

Marquis et al. examine the causal attributions of poverty (individual or social blame and individual or social fate) and how these views relate to socio-economic stratification. They adopt a broad perspective on social stratification—covering education, income, wealth, deprivation, mobility, and class—and show that self-interest and self-serving bias shape poverty attributions: higher social positions align with greater individual and fewer social blame attributions. Yet higher education is linked to social blame, and these attributions remain stable in the short term despite shifts in social stratification.

Hadziabdic examines whether active participation in voluntary associations can reduce the political participation gap by educational level. He shows that the highly educated are more likely to be involved in associations and be more politically engaged, and active involvement in associations boosts political engagement across the board, especially for the less educated, who typically start with lower levels of political activity. He concludes that associations thus help narrow political participation gaps based on educational level, particularly in areas like party membership and local volunteering.

Taken together, the collection of studies paints a nuanced but consistent picture. Despite educational expansion and structural economic changes, social stratification remains deeply embedded in individual life courses. Education is both a mechanism of mobility and a reproducer of inequality. Across decades and domains—from education to labor market entry, occupational prestige, and even attitudes toward poverty—social origin continues to shape individual life chances in profound ways.

